# Effect of Lanthanum Sorption on the Behavior of Rarely Crosslinked Acidic and Basic Polymer Hydrogels during Remote Interaction

**DOI:** 10.3390/polym15061420

**Published:** 2023-03-13

**Authors:** Yevgeniy Melnikov, Ruslan Kondaurov, Laura Agibayeva

**Affiliations:** 1 Biochemical Engineering Department, International Engineering and Technological University, Al-Farabi Ave. 93a, 050060 Almaty, Kazakhstan; 2 Faculty of Chemistry and Chemical Technology, Al-Farabi Kazakh National University, Al-Farabi Ave. 71, 050040 Almaty, Kazakhstan

**Keywords:** lanthanum, sorption, separation, rarely crosslinked polymeric hydrogels, interpolymer systems, remote interaction

## Abstract

This present study is targeted at the complex investigation of the behavior of interpolymer systems based on acidic rarely crosslinked polymeric hydrogels (polyacrylic acid hydrogel (hPAA); polymethacrylic acid hydrogel (hPMAA)) and basic rarely crosslinked polymeric hydrogels (poly-4-vinylpyridine hydrogel (hP4VP), specifically the poly-2-methyl-5-vinylpyridine hydrogel (hP2M5VP)) either in an aqueous medium or lanthanum nitrate solution. We found that the transition of the polymeric hydrogels in the developed interpolymer systems (hPAA-hP4VP, hPMAA-hP4VP, hPAA-hP2M5VP, and hPMAA-hP2M5VP) into highly ionized states leads to significant changes in electrochemical, conformational, and sorption properties of the initial macromolecules. The subsequent mutual activation effect demonstrates strong swelling of both hydrogels in the systems. The sorption efficiency of lanthanum by the interpolymer systems is 94.51% (33%hPAA:67%hP4VP), 90.80% (17%hPMAA-83%hP4VP), 91.55% (67%hPAA:33%hP2M5VP), and 90.10% (50%hPMAA:50%hP2M5VP). An advantage of the interpolymer systems (compared to individual polymeric hydrogels) is the strong growth of their sorption properties (up to 35%) due to high ionization states. Interpolymer systems can be considered new-generation sorbents for further application in the industry for highly effective sorption of rare earth metals.

## 1. Introduction

The constant increase in rare earth metals (REMs) demand is associated with a variety of areas of their application. REMs have a significant role in the production of high-tech consumption areas materials involved in electronic/electro-optical industries, information technology, biomedicine, environmental protection, and energy saving. They are also used in the production of phosphors, industrial ceramics, oil refining and automotive industries catalysts, high-quality glass, permanent magnets, superconductors, fiber optics, oxygen sensors, lasers, long-life batteries for electric vehicles, and film and photographic equipment. In addition, wide usage of REM is observed in traditional areas of consumption, particularly metallurgy [1,2,3,4,5,6,7,8,9,10].

In Kazakhstan, work has been carried out to study the elemental composition and assess the geochemical spectrum of more than 30 deposits [11]. The results obtained indicate that uranium deposits can be characterized by high content of rare metals (RM), REM, and gold. They predominantly accumulate light lanthanides, in particular lanthanum and cerium.

Kazakhstan can be considered one of the world’s largest regions with considerable reserves and further prospects for expanding the mineral-resource base (for example, REM and RM). In the Republic of Kazakhstan, REM/RM production occurs at specialized industrial enterprises (these metals are produced as by-products at non-ferrous metallurgical enterprises). Nowadays, REM/RM production, along with their compounds in the Republic, can be characterized as highly unstable (in other words, far from meeting its potential) [12].

In enterprises aimed at REM/RM production, there is no progress in expanding the range of products due to the loss of the Kazakhstani niche in the world metal market, deterioration in the quality of products, lack of stable raw materials sources, outdated technology and equipment, and lack of funding. These and other factors have led the sub-sector to a virtual stagnation [13,14,15].

From the point of view of the world’s requirements for the development of science along with technology based on the increasing demand for rare/rare-earth products, the production of REM/RM can be described as highly profitable. Highly developed countries have advanced technologies for technical purity metals refining produce products for further use in the following areas: electronics; radio/electrical engineering along with the rest of high-tech industries for further application in space aviation; and instrument-building technology [16,17,18,19,20,21,22,23,24].

Based on the above-mentioned, it can be concluded that the main priority for the Republic of Kazakhstan is the final production of high-purity REM/RM along with their compounds. The noted REM/RM production will provide the development of electronics, instrument-making, and semiconductors, along with other prospective areas of science/technology. Moreover, almost all developed or explored reserves of mineral ores in Kazakhstan contain REM/RM [25,26,27,28,29,30].

Individual REM is in the greatest demand on the world market. Difficulties lie in the separation of a certain element from its sum and the paucity of profitable REM deposits. Therefore, it is necessary to create a technology for obtaining a wide range of individual rare earth elements. For the concentration, separation, and purification of REM from impurities, various methods are used, including extraction and ion exchange [31,32,33,34,35,36,37,38,39].

Modern sorption technologies are based on the application of ion exchangers. These sorbents seem to be promising, except that they are selective to a certain metal (sorption of each REM requires a certain ion exchanger), and full regeneration of the sorption capacity to the manufacturing values is rather long and sometimes complicated. As an alternative to the mentioned ion-exchange sorbents, it is supposed to use interpolymer systems consisting of two rarely crosslinked polymer hydrogels for the sorption of REM. The novelty of this scientific study is the application of the remote interaction effect for high ionization of the two initial components of the interpolymer systems for the further significant growth of sorption parameters. This investigation aims to establish the possibility of interpolymer systems application for REM efficient sorption (on the example of lanthanum sorption).

## 2. Materials and Methods

### 2.1. Materials

Monomers: acrylic acid (AA) (purity 99%) and methacrylic acid (MAA) (purity 99%) (Sigma-Aldrich; Rockville, MD, USA). Linear polymers: poly-4-vinylpyridine (P4VP) and poly-2-methyl-5-vinylpyridine (P2M5VP) (Reakhim; Moscow, Russia). Crosslinkers: N,N-methylene-bis-acrylamide (MBA) (purity 99.0%) and epichlorohydrin (ECH) (purity 99.0%) (Sigma-Aldrich; Rockville, MD, USA). Initiator: potassium persulfate (purity ≥ 99.0%) (Reakhim; Moscow, Russia). Solvent: dimethylformamide (DMF) (purity ≥ 99.0%) (Reakhim; Moscow, Russia). Salt: lanthanum nitrate hexahydrate (purity 99.9%) (Reakhim; Moscow, Russia).

Preliminary step: before using the monomers, AA and MAA underwent vacuum distillation for purification from MEHQ inhibitor.

### 2.2. Methods

#### 2.2.1. Synthesis of Rarely Crosslinked Polymeric Hydrogels

##### Acidic Hydrogels Synthesis

For the synthesis of acidic hydrogels (PAA/PMAA), the following synthesis technique was used: AA/MAA monomer (10 mL) is added volumetric flask (100 mL); subsequently, water (40 mL) is added, and after which the crosslinker MBA was added with the final step adding of the reaction initiator potassium persulfate (1 mL). The reactive mixture in the flask was brought up to the mark with distilled water. Then, pour the polymerizate into an ampoule before placing it in a drying cabinet, heated to 60–65 °C. Low concentration of the crosslinker in the polymerizate provides rare crosslink distribution along the polymer chain. Yield of the polymerization process is 65.94% for PAA hydrogel and 62.21% for PMAA hydrogel. Polymerization time in both cases was 25 min.

##### Basic Hydrogels Synthesis

The synthesis procedure of the basic hydrogels (P4VP/P2M5VP) was carried out as follows: P4VP/P2M5VP linear polymer portion (5 g) is poured by DMF (15 mL) to complete dissolving of the polymer (includes polymer swelling stage). Then, add crosslinker ECH (2 mL) and continuously stir at temperature 65 °C. Yield of the polymerization process is 84.27% for P4VP hydrogel and 79.89% for P2M5VP hydrogel. Polymerization time in both cases was 30 min.

##### Purification and Sampling of the Synthesized Rarely Crosslinked Polymeric Hydrogels

Purification process of the synthesized polymeric hydrogels (hPAA, hPMAA, hP4VP, and hP2M5VP) from unreacted impurities and the polymers soluble fraction was carried out by prolonged washing with distilled water (under stationary room conditions at 3 times daily water change). The process of hydrogel purification was controlled by measuring washing water conductivity/pH. After 2 weeks (14 days), the values of these parameters of washing water remained permanent, which points to the completion of the purification process. Subsequently, the polymeric hydrogels were placed for drying. Then, the resulting hydrogel samples were ground to a dispersion (the size of polymeric granules: 130 ≤ d ≤ 150 µm). After above procedures, the polymeric hydrogels are ready for experiments.

Swelling degree of the purified hPAA, hPMAA, hP4VP, and hP2M5VP is 39.34 g/g, 27.16 g/g, 3.53 g/g, and 3.18 g/g, respectively.

#### 2.2.2. Preparation of Interpolymer Systems

The special cells made from polypropylene (pore sizes of the material are no more than 100 µm) were used for placing the synthetized macromolecular dispersions. The necessary application of these cells is through remote interaction in the interpolymer systems due to the impermeability of hydrogels’ dispersion and permeability of metal ions. Subsequently, the polypropylene cells are placed in the lanthanum nitrate solution; in the case of the interpolymer pairs, distance between the cells is 2.5 cm. Total amount of the polymers in the solution is 100 mol.% (for individual hydrogels in interpolymer pairs). The following interpolymer systems were prepared: hPAA-hP4VP; hPMAA-hP4VP; hPAA-hP2M5VP; and hPMAA-hP2M5VP.

#### 2.2.3. Sorption Experiments

For the experiments, lanthanum nitrate hexahydrate salt solution was prepared (concentration: 100 mg/L). The dispersion of polymeric hydrogels was put into the salt solution for 2 days (48 h). Sampling of aliquotes occurred at a certain time.

#### 2.2.4. Equipment

Electric conductivity was measured via using conductometer Expert 002-2-6p (Econics-expert; Moscow, Russia). pH-meter 780 Metrohm (Metrohm; Herizau, Switzerland) was used for measuring solutions pH values. Hydrogels mass was measured on Shimadzu AY220 analytical balance (Shimadzu, Japan). Residual concentration of lanthanum was calculated via measurement of the solution optical density on photocolorimeter KFK-3-01 (ZOMZ; Sergiyev Posad, Russia). Additionally, concentration was measured via Optima 8300 ICP-OES spectrometer (Perkin-Elmer; Waltham, MA, USA).

#### 2.2.5. Calculation of the Parameters

Calculation of polymerization yield:

(1) $$polymerization\;yield=\frac{m_{p}}{m_{r}}*100\%$$

where *m_p_* is the weight of the synthetized polymeric hydrogel after purification, g, and *m_r_* is the weight of the initial reactants (monomers/linear polymers), g.

Calculation of polymeric hydrogels swelling degree:

(2) $$\alpha =\frac{m_{2}-m_{1}}{m_{1}}$$

where *m*_1_ is the weight of the dry polymeric hydrogel, g, and *m*_2_ is the weight of the swollen polymeric hydrogel, g.

Extraction degree of lanthanum is expressed as follows:

(3) $$\eta =\frac{C_{0}-C_{r}}{C_{0}}*100\%$$

where *C*_0_ is the initial La concentration, mg/L, and *C_r_* is the residual La concentration, mg/L.

Dynamic exchange capacity is expressed as follows:

(4) $$Q=\frac{\nu_{sorbed}}{m_{sorbent}}$$

where *m_sorbed_* is the sorbed lanthanum amount, mmol, and *m* is the macromolecule’s portion mass (in case when two polymers in the solution (interpolymer pair), the mass is equal to sum of each polymeric hydrogel mass), g.

## 3. Results and Discussion

The sorption of the lanthanum by the developed interpolymer systems leads to significant changes in the polymeric hydrogels initial properties in comparison with their behavior in an aqueous medium (e.g., distilled water).

### 3.1. Differences of the Interpolymer Systems Behavior in Dependence from Reactive Media

**Figure 1:**
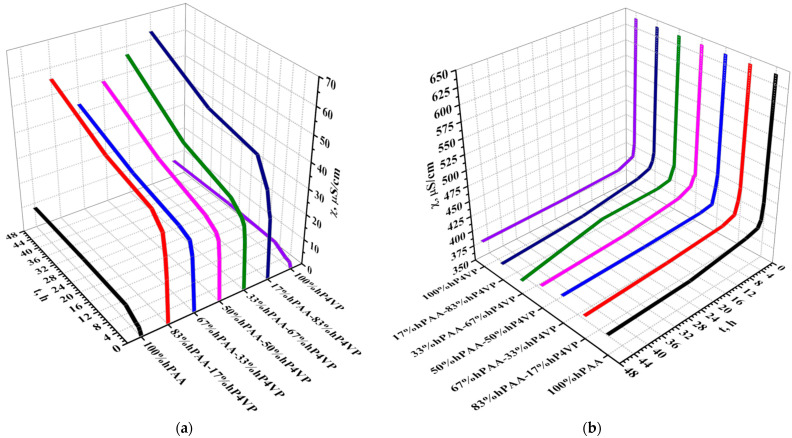

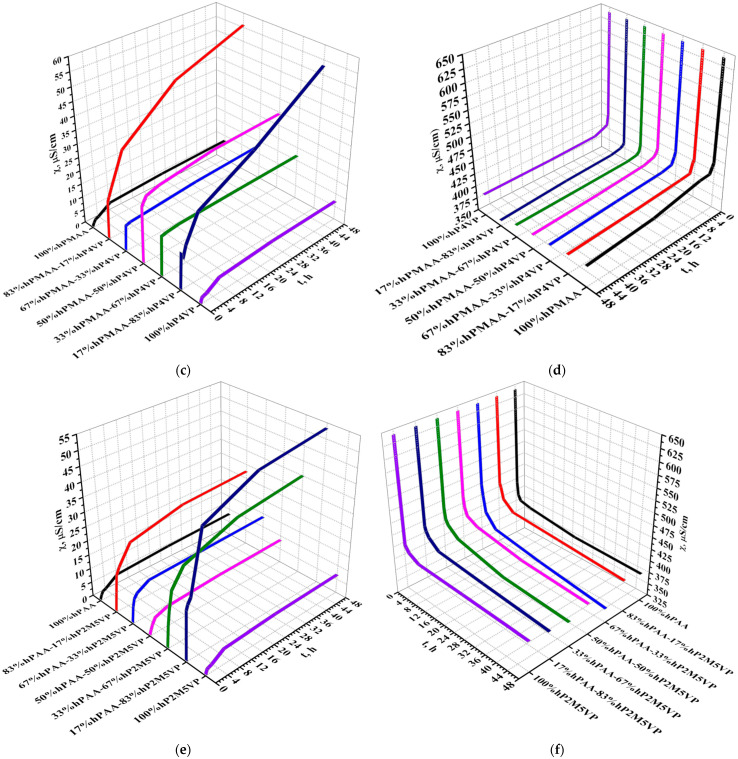

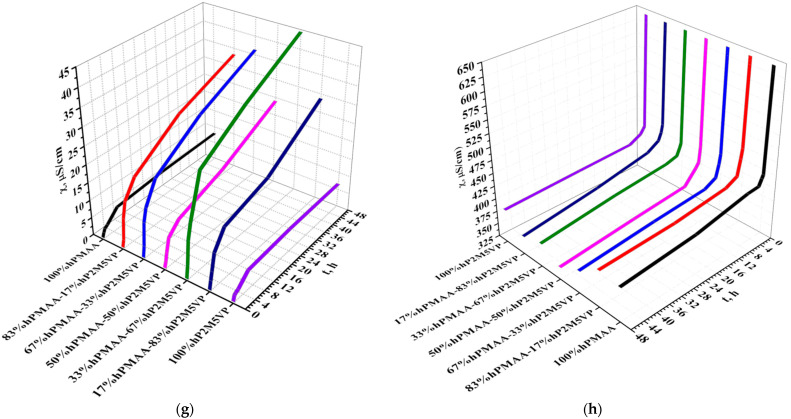
Electric conductivity of water (**a**,**c**,**e**,**g**) and lanthanum nitrate (**b**,**d**,**f**,**h**) in presence of interpolymer systems.

Figure 1 shows specific electrical conductivity in the water medium (a,c,e,g) and lanthanum nitrate (b,d,f,h) in the presence of the interpolymer systems (hPAA-hP4VP, hPMAA-hP4VP, hPAA-hP2M5VP, and hPMAA-hP2M5VP). In the process of remote interactions of polymeric hydrogels in the interpolymer systems, there is an occurrence of aqueous solutions with specific electrical conductivity changes. The increase of water electrical conductivity with time was observed for all polymeric hydrogel ratios in the interpolymer systems. Nevertheless, electrical conductivity changes are rather diverse for various molar ratios of polymeric hydrogels. It should be noted that regions of maximum and minimum electrical conductivity can be observed. The minimum electrical conductivity can be seen in the presence of individual polymeric hydrogel dispersions (polyacid/polybasis). This is a consequence of the absence of the mutual activation effect of interacting polymeric structures, which, in turn, causes a rather quick reaching of electrochemical equilibrium in the solution. It is evidenced by the fact that the electrical conductivity changes very slightly after 6 h of interaction with the polymer structures with an aqueous solution. Maximum electrical conductivity in the interpolymer systems is achieved after 48 h at 17%hPAA:83%hP4VP, 83%hPMAA:17%hP4VP, 17%hPAA:83%hP2M5VP, and 33%hPMAA:67%hP2M5VP molar ratios. Polymeric hydrogel electrochemical properties significantly increase in the interpolymer systems occurrence owing to the mutual activation effect, which leads to the transition of the polymeric hydrogels into highly ionized states. However, it is impossible to judge the degree of mutual activation only by electrical conductivity because increased values may indicate the dominance of the process of dissociation of carboxylic groups over the association of a proton by the heteroatoms of basic polymeric hydrogels.

The behavior of the intepolymer systems in the lanthanum nitrate solution is different in comparison with the behavior in the water medium. The obtained data shows the decrease of electrical conductivity for all polymeric hydrogel ratios in the developed interpolymer systems with time. The main reason for the decrease in the parameter is the sorption of lanthanum. Based on the obtained results of electric conductivity, the process of lanthanum sorption can be described as follows: at first, the remote interaction between acidic and basic polymeric hydrogels occurs, resulting in proton release owing to the carboxylic (functional) groups dissociation. Contact of the rarely crosslinked polymeric hydrogels in the salt solution leads to a decrease of conductivity from REM sorption, wherein a significant decrease is observed 30 min after the beginning of each interpolymer system’s contact with the REM salt. Further interaction between the rarely crosslinked macromolecules provides a consequent decrease in conductivity. It should be noted that, in addition to formed reactive groups and particles (–COO^−^ groups and H^+^ ions), the solution contains products of dissociation of lanthanum nitrate. With the mutual activation of polymer hydrogels in the interpolymer systems, dissociation products are released into the solution; however, electrical conductivity decreases, which indicates the binding of the rare earth metal by the polymers.

**Figure 2:**
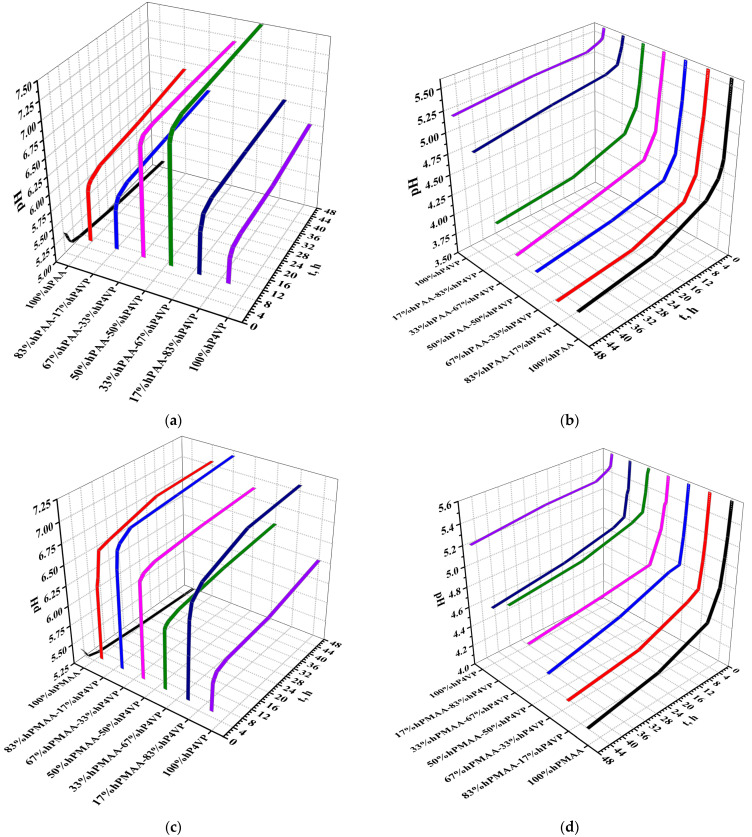

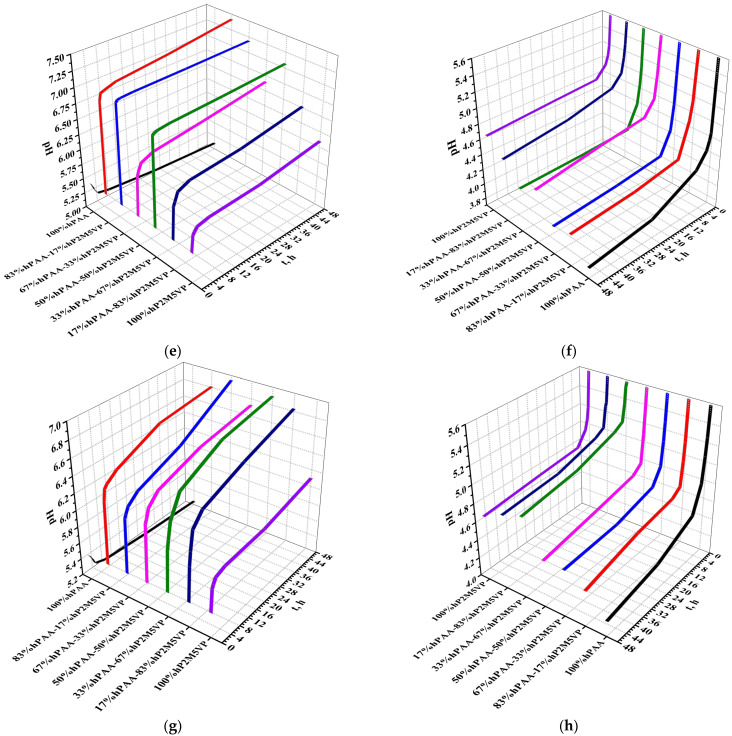
pH of water (**a**,**c**,**e**,**g**) and lanthanum nitrate (**b**,**d**,**f**,**h**) in presence of interpolymer systems.

Figure 2 shows the pH of water (a,c,e,g) and lanthanum nitrate (b,d,f,h) in the presence of interpolymer systems. The obtained data show that pH increases in the interpolymer systems over time, which, in turn, indicates to ongoing processes of ionization of polymeric hydrogels. The obtained data on pH increase show that hydrogen ions (formed during dissociation of carboxylic groups) are bound by heteroatoms of the vinylpyridine’s ring. Values of pH maximums can point to the fact that the dissociation rate of the carboxylic groups is slower compared to the process of vinylpyridine’s nitrogen atom protonization. In other words, it can be said that mutual activation of the macromolecular rare crosslinked structures in the interpolymer systems provides the formation of similar charges (without counter-ions) on the internodal links on the polymeric chains. If these data will be compared with the data on electrical conductivity, it can be concluded that at certain ratios (where low conductivity values and high pH values), the process of proton addition by vinylpyridine prevails over the process of dissociation of carboxyl groups. The decrease in pH occurs in the presence of only an acidic hydrogel. This is a consequence of the release of protons into the solution as a result of electrolytic dissociation of carboxylic groups, which provides an increase in H^+^ ions concentration.

Lanthanum nitrate in the presence of interpolymer systems observed a pH decrease over time. This phenomenon can be explained as follows: ionization of basic polymeric hydrogels can occur not only due to the association of protons but the formation of coordination bonds with lanthanum nitrate dissociation products (in other words, sorption of lanthanum) as well. Due to the binding of lanthanum by the carboxylate anions of the acidic polymer hydrogels, their solution amount decreases, which, in turn, leads to the formation of additionally dissociated carboxylic groups, resulting in a strong growth of sorption properties of acidic hydrogel in the interpolymer pair.

**Figure 3:**
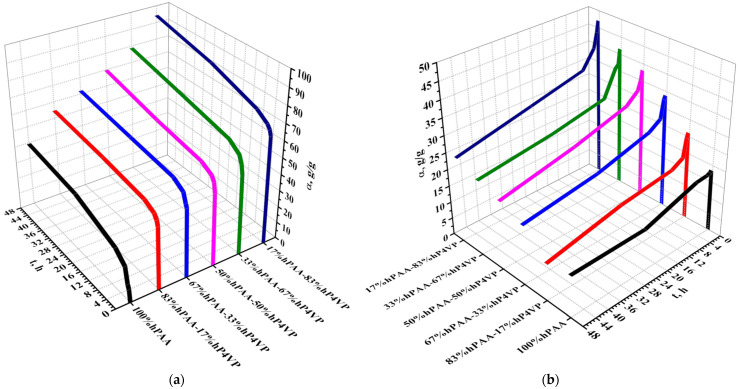

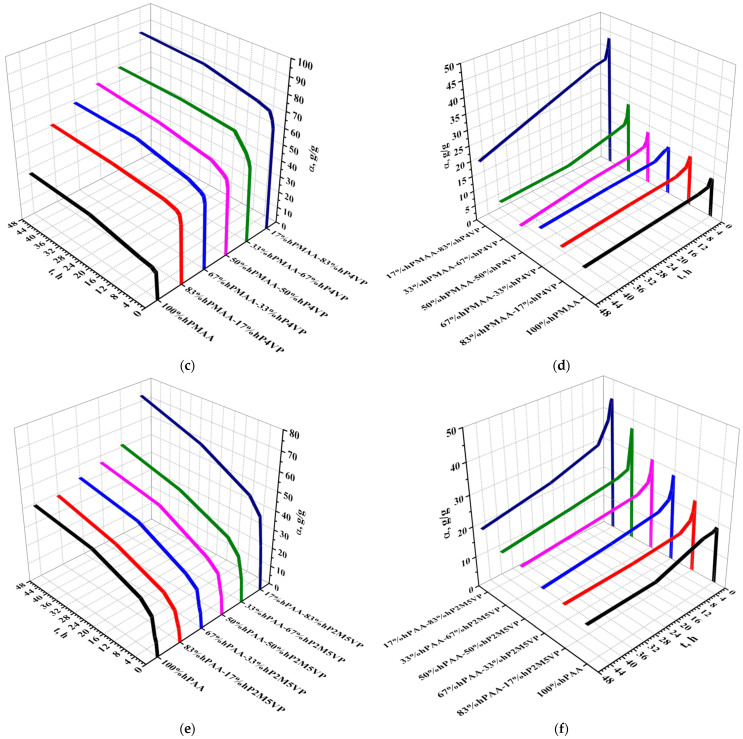

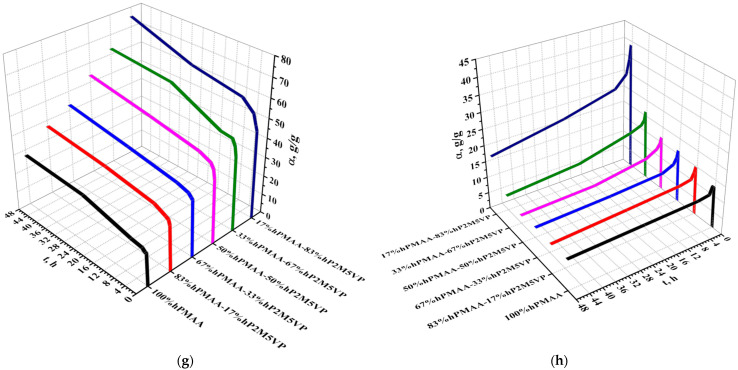
Swelling degree of acidic hydrogels in aqueous medium (**a**,**c**,**e**,**g**) and lanthanum nitrate (**b**,**d**,**f**,**h**).

**Figure 4:**
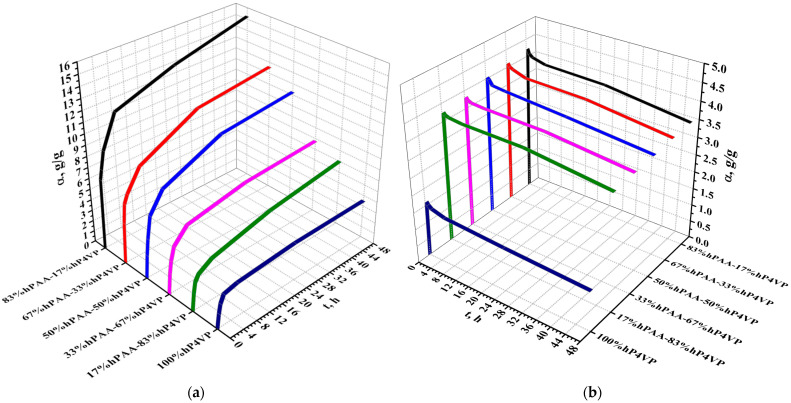

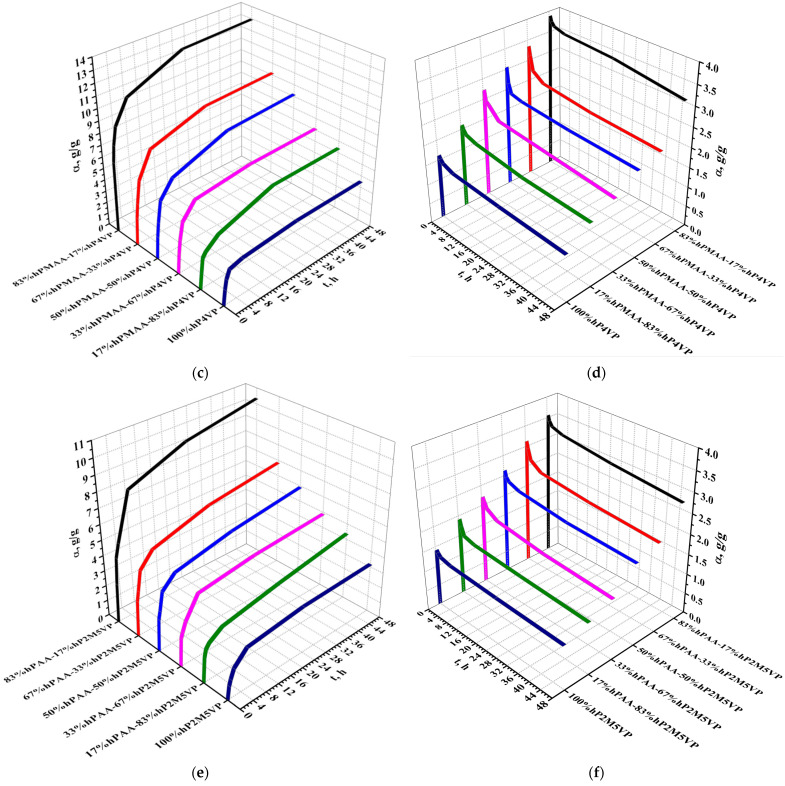

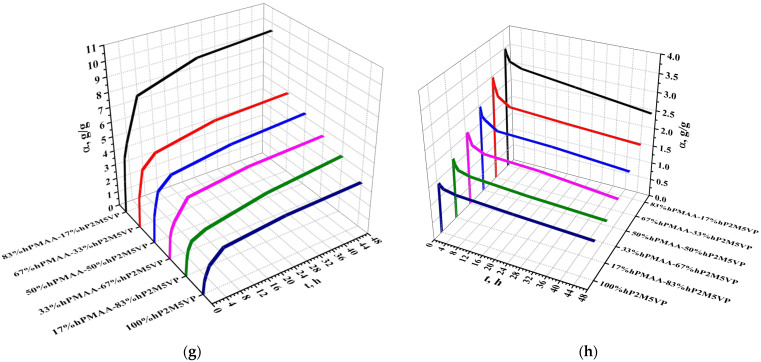
Swelling degree of basic hydrogels in aqueous medium (**a**,**c**,**e**,**g**) and lanthanum nitrate (**b**,**d**,**f**,**h**).

The swelling degree of acidic polymeric hydrogels (hPAA and hPMAA) in different interpolymer systems in dependence on solution origin (water or lanthanum nitrate) is shown in Figure 3. The swelling degree of the polyacids increases with time in aqueous media. Additionally, the strong growth of swelling is seen when the share of polybasis increases in the interpolymer systems. Such increase points to the predominance of the process of protons association over the process of carboxyl groups dissociation. The mutual activation of the hydrogels and subsequent transition into highly ionized states provide the formation of same-charged carboxylate ions on internodal links of the polyacids leading to the unfolding of the polymeric globe. Minimum swelling of the acidic polymeric hydrogels occurs due to the absence of a mutual activation effect, wherein equilibrium between the polymers and aqueous medium is reached quickly (after 6 h increase of swelling degree is slight; after 24 h, almost insignificant). Swelling in the salt solution is different from swelling in water, as seen from Figure 4b,d,f,h. The initial swelling degree increase (during first 30 min) for polyacids occurs due to a mutual activation; further lanthanum sorption provides a decrease in the swelling degree. The sorption of lanthanum leads to a state in which the polymers do not have same-charged groups (leading to polymer globe unfolding), the result of which is a decrease in swelling due to the folding of the macromolecular globe. The same tendency is observed in the case of polyacids swelling in water: an increase in the polybasis share leads to an increase in the swelling of polyacid.

The swelling degree of basic hydrogels (hP4VP, hP2M5VP) in a water medium (a,c,e,g) and lanthanum nitrate (b,d,f,h) is shown in Figure 4. Similar to the swelling of polyacids, the increase in the swelling degree of the polybases in aqueous media is observed with time and the growth of another component (polyacid) share. This increase of swelling indicates the additional ionization of links of the polybases from the mutual activation of both acidic and basic hydrogels in interpolymer systems and from the additional release of hydrogen ions in an aqueous medium during the remote interaction of polymers. It should be noted that the increase in polyacid shares leads to a shift of the equilibrium to the right (to the additional dissociation of carboxyl groups). The swelling increase, in this case, can be described as follows: the main increase in the degree of swelling occurs within 2 h, after which the parameter changes slightly, indicating that the system polymer–water is approaching an equilibrium state. The swelling of the polybases in the lanthanum nitrate solution has another characteristic compared to the swelling in an aqueous medium. The initial increase of swelling is associated with the rarely crosslinked polymeric hydrogels’ mutual activation effect, which transfers the macromolecules into highly ionized states. Afterwards, there is a decrease in swelling due to the folding of the macromolecular globe, wherein maximum swelling is observed at 30 min of the interaction. This is due to the release of large amounts of protons by the polyacid and their binding of nitrogen heteroatoms to the polybases, after which there is a gradual decrease in swelling. The lowest swelling of the polybases occurs in the presence of individual basic polymeric hydrogels (hP4VP and hP2M5VP). The absence of the mutual activation phenomenon when only polybases are present in the salt solution leads to a minimal degree of swelling of basic polymeric hydrogels. The impossibility of polybases transitioning to highly ionized states is due to the fact that there is no additional activation of units that promotes the unfolding of the macromolecular coil. Lanthanum sorption results in polymeric hydrogels folding and a decrease in the degree of polybases swelling over time.

Based on the obtained results on electrical conductivity, pH, and swelling degree of the interacting rarely crosslinked macromolecules, it could be said that the concentration of H^+^ ions in the interpolymer systems determines the equilibrium in two processes:

(1) Through the swelling rates, at which H^+^ ions are released in solution as a result of dissociation of –COOH groups;
(2) Through the binding rates of H^+^ ions by polybase units.

### 3.2. Sorption Parameters of the Interpolymer Systems in Relation to Lanthanum Ions

**Figure 5:**
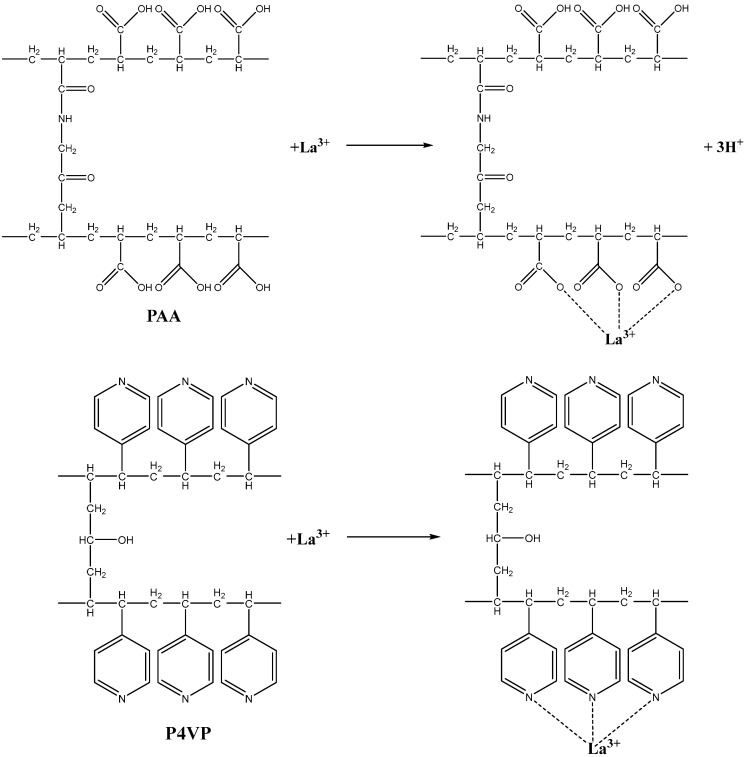
Proposed mechanism of lanthanum sorption for PAA and P4VP hydrogels.

The highly ionized state of the rarely crosslinked macromolecules in the interpolymer systems leads to significant increase in sorption properties compared to individual polymeric hydrogels. The proposed mechanism of the lanthanum sorption by the acidic and basic hydrogels is presented in Figure 5.

**Figure 6:**
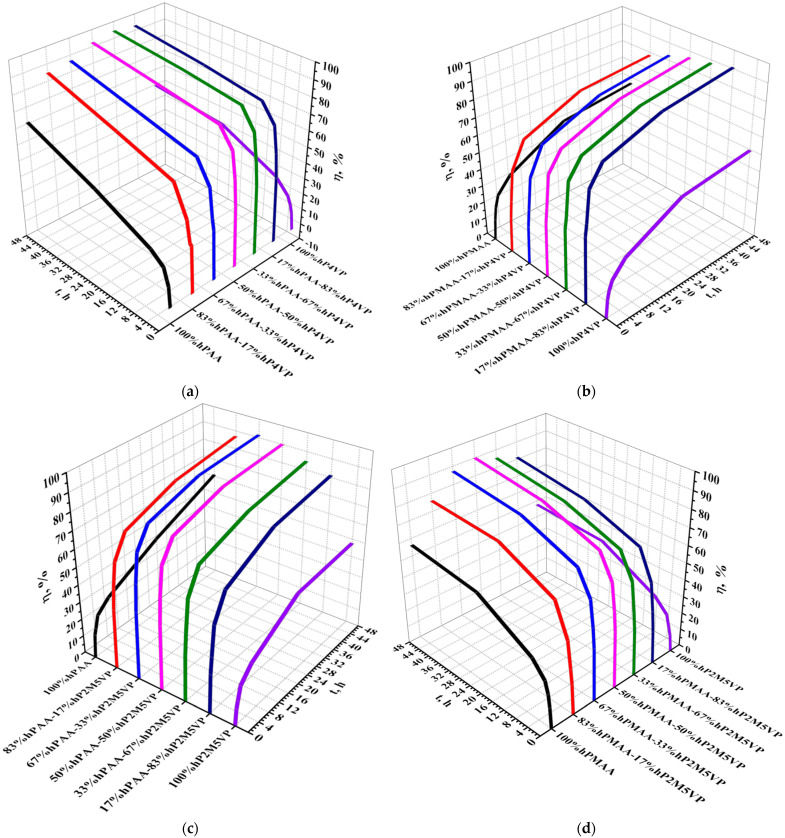
Extraction degree of lanthanum of the interpolymer systems hPAA-hP4VP (**a**), hPMAA-hP4VP (**b**), hPAA-hP2M5VP (**c**), and hPMAA-hP2M5VP (**d**).

Figure 6 presents the extraction degree of lanthanum in the interpolymer systems (hPAA-hP4VP (a), hPMAA-hP4VP (b), hPAA-hP2M5VP (c), hPMAA-hP2M5VP (d)). The obtained data shows that interpolymer pairs have higher sorption degree values (in comparison with individual polymeric hydrogels). The sorption degree of lanthanum for individual rarely crosslinked polymeric hydrogels (hPAA, hPMAA, hP4VP, and hP2M5VP) does not exceed 61–60.94% (hPAA), 56.34% (hPMAA), 49.54% (hP4VP), and 47.74% (hP2M5VP) for the interacting time of 48 h. The reason for such low values of extraction degrees is the sufficiently rapid achievement of equilibrium in solutions between the present macromolecules and dissociated ions of rare earth metal. In the interpolymer systems, not intense sorption occurs at ratios 83%hPAA:17%hP4VP (for system hPAA-hP4VP), 83%hPMAA:17%hP4VP (for system hPMAA-hP4VP), 17%hPAA:83%hP2M5VP (for system hPAA-hP2M5VP), and 83%hPMAA:17%hP2M5VP (for system hPMAA-hP2M5VP), which indicates the insufficient ionization degrees of these hydrogels in the interpolymer systems. The highest ionization degree of the polymeric hydrogels is observed at ratios 33%hPAA:67%hP4VP (for system hPAA-hP4VP), 17%hPMAA:83%hP4VP (for system hPMAA-hP4VP), 67%hPAA:33%hP2M5VP (for system hPAA-hP2M5VP), and 50%hPMAA:50%hP2M5VP (hPMAA-hP2M5VP), which is evidenced by the maximum values of the extraction degree (94.51%, 90.80%, 91.55%, and 90.10%, respectively) at 48 h of remote interaction. At these ratios, optimal conformation for lanthanum sorption is formed in the studied interpolymer systems.

The values of the lanthanum sorption degrees of the studied interpolymer systems are presented in Table 1.

**Figure 7:**
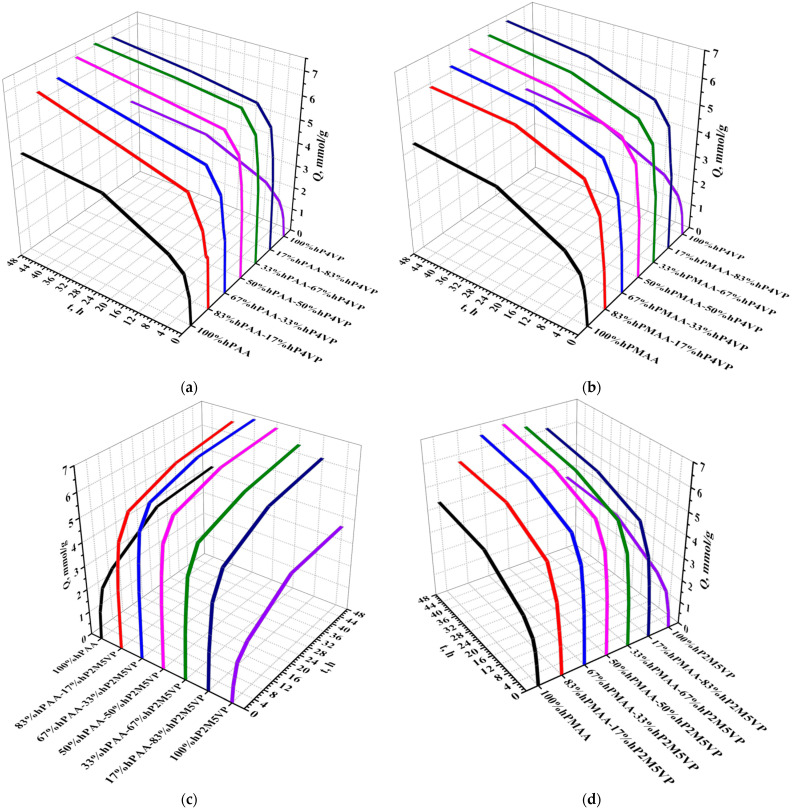
Dynamic exchange capacity (in relation to La) of the interpolymer systems hPAA-hP4VP (**a**), hPMAA-hP4VP (**b**), hPAA-hP2M5VP (**c**), and hPMAA-hP2M5VP (**d**).

Figure 7 represents the dynamic exchange capacity (in relation to La) of the studied interpolymer systems (hPAA-hP4VP (a), hPMAA-hP4VP (b), hPAA-hP2M5VP (c), and hPMAA-hP2M5VP (d)). Interpolymer pairs have a much higher exchange capacity compared to individual hydrogels due to the fact that initial macromolecules are transferred into highly ionized states owing to the mutual activation effect. The maximum exchange capacity is observed at ratios 33%hPAA:67%hP4VP (for system hPAA-hP4VP), 17%hPMAA:83%hP4VP (for system hPMAA-hP4VP), 67%hPAA:33%hP2M5VP (for system hPAA-hP2M5VP), and 50%hPMAA:50%hP2M5VP (for system hPMAA-hP2M5VP), which points to high ionization of the macromolecules in these interpolymer pairs. It should be noted that the growth of exchange capacity at the mentioned molar ratios is over 30% in comparison with individual polymeric hydrogels. The exchange capacity’s lowest values in the interpolymer systems are observed at ratios 83%hPAA:17%hP4VP (for system hPAA-hP4VP), 83%hPMAA:17%hP4VP (for system hPMAA-hP4VP), 17%hPAA:83%hP2M5VP (for system hPAA-hP2M5VP), and 83%hPMAA:17%hP2M5VP (for system hPMAA-hP2M5VP), which indicate that ionization degrees of the initial polymeric hydrogels at these ratios are insufficiently high. The low values of exchange capacity in the presence of individual hydrogels (hPAA, hPMAA, hP4VP, and hP2M5VP) due to the absence of the mutual activation phenomenon and subsequent transition into highly ionized states.

The values of the dynamic exchange capacity (in relation to La) of the studied interpolymer systems are presented in Table 2.

### 3.3. IR Spectra of Rarely Crosslinked Polymeric Hydrogels before/after Lanthanum Sorption

For the IR spectra comparison of the obtained polymer complexes of rare crosslinked polymeric hydrogels, PAA, PMAA, P4VP, and P2M5VP, with lanthanum ions and the spectra of the initial hydrogels (without sorbed lanthanum ions), the following molar ratios were selected (due to the fact that maximum lanthanum sorption occurs at these ratios): 33%hPAA-67%hP4VP, 17%hPMAA-83%hP4VP, 67%hPAA-33%hP2M5VP, and 50%hPMAA-50%hP2M5VP.

#### 3.3.1. Characterization of the IR Spectra of Hydrogels PAA/P4VP before/after Lanthanum Sorption by the 33%hPAA-67%hP4VP Interpolymer System

**Figure 8:**
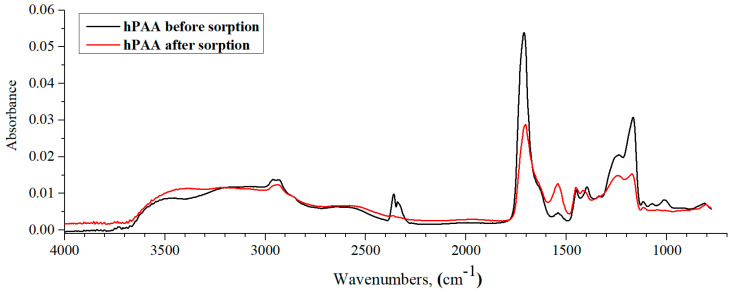
IR spectra of hPAA before and after lanthanum sorption by interpolymer system 33%hPAA-67%hP4VP.

Figure 8 shows the IR spectra of hPAA before and after lanthanum sorption by the studied interpolymer system. From the figure, one can observe that the absorption band intensity decreases in the 1700 cm^−1^ region (responsible for the stretching vibrations of the C=O group) by almost 2 times. In this case, it must be taken into account that the carboxyl group is in an ionized state, i.e., in the polymer, this group is present as a carboxylate anion. In the process of sorption, lanthanum ions, due to the electrostatic interaction with the carboxylate anion, reduce the charge on the polymer, which ultimately leads to an absorption band intensity decrease. Additionally, in the process of charge loss on the carboxyl group, the electron density is redistributed, which is reflected in the absorption band intensity decrease in the 1300–1120 cm^−1^ region (C-O stretching vibrations in the carboxylic group). At the same time, practically no changes are observed in the spectrum of the polymer after sorption in the absorption region 1440–1380 cm^−1^, which is responsible for the CH–CO group. The absorption region 3600–2900 cm^−1^ also remained unchanged, in which intramolecular bonds of the polymer manifested hydrogen bonds between OH groups in the composition of the carboxyl group of the polymer. It is worth noting here that some parts of the polymeric hydrogel’s carboxylic groups are in an uncharged state, which in turn leads to hydrogen bond formations between them and further inability to participate in the sorption process.

Thus, based on the obtained IR spectra, it can be concluded that in the case of polyacrylic acid, the charged carboxyl group of the polymer participates in the process of lanthanum sorption.

**Figure 9:**
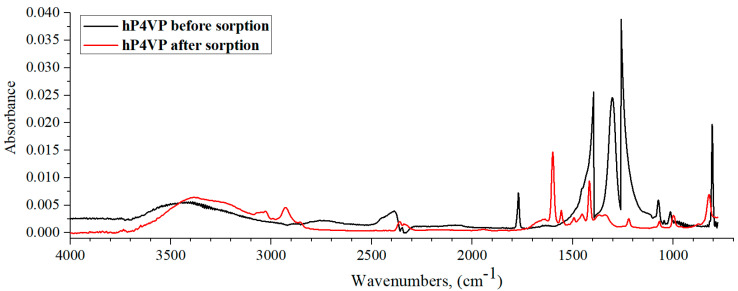
IR spectra of hP4VP before and after lanthanum sorption by interpolymer system 33%hPAA-67%hP4VP.

Figure 9 shows the IR spectra of hP4VP before and after lanthanum sorption by the studied interpolymer system. The polybasis’ IR spectrum after La sorption is characterized by some absorption band intensities decreasing to the noise level, in which new characteristic frequency formations occur. So, before lanthanum sorption, intense absorption bands on the hP4VP IR spectrum can be seen in the so-called “fingerprint” region—1400, 1300, and 1250 cm^−1^ (the mentioned regions correspond to the deformation vibrations of -CH- and -CH_2_-groups of the polymeric hydrogel’s main chain, which are absent in the same polymeric hydrogel IR spectrum after La sorption). We can also note the appearance of the characteristic band at 1600 cm^−1^ in the polybasis’ spectrum after sorption, which is responsible for the C=N group. Additionally, from this obtained data, on the spectrum of the polymer before sorption, one can observe an absorption band of medium intensity in the region of 2500–2350 cm^−1^, which characterizes the R_2_C=NH^+^ group. Hence, it can be said that the polymer is in a charged state before sorption in solution, even though P4VP hydrogel has a low degree of ionization. However, after sorption, this absorption band loses its intensity and shifts to the region of 2400–2320 cm^−1^, which indicates a partial loss of the charge of the polymer chain during the sorption of lanthanum ions. From all this data, it follows that in the process of sorption, P4VP hydrogel is deionized, followed by the formation of a coordination complex with lanthanum ions through the nitrogen atom. In turn, the electron-donating properties of the nitrogen atom are enhanced, especially in combination with double-bond conjugation to the carbon atom.

#### 3.3.2. Characterization of the IR Spectra of Hydrogels PMAA/P4VP before/after Lanthanum Sorption by the 17%hPMAA-83%hP4VP Interpolymer System

**Figure 10:**
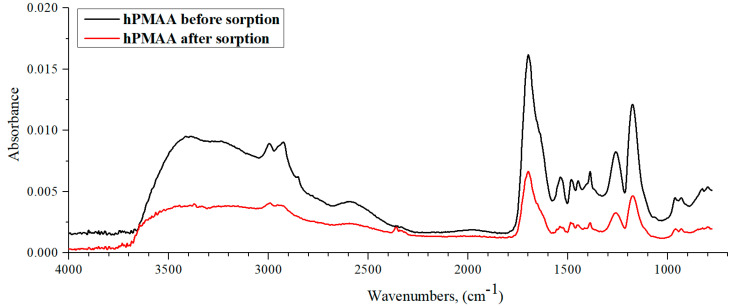
IR spectra of hPMAA before and after lanthanum sorption by interpolymer system 17%hPMAA-83%hP4VP.

Figure 10 shows the IR spectra of hPMAA before and tafter lanthanum sorption by the studied interpolymer system. It can be said that the hPMAA spectrum after La sorption is almost identical to the original spectrum (only the absorption band’s intensity decreases over 3 times). The intensity decrease points to La sorption. As in the case of PAA hydrogel, PMAA hydrogel is also characterized by the mechanism of metal ions’ sorption through the carboxylic group, which is pointed out by the –COOH group absorption band intensity decrease in the region of 1700 cm^−1^. However, it should be noted that the decrease in intensity is more noticeable than in the case of PAA owing to the impact of the CH_3_ substituent on the hPMAA chain stretching vibrations. In addition, the substituent influences hydrogen bond formations between the carboxylic group’s -OH fragments. Compared with PAA rarely crosslinked polymeric hydrogel (Figure 8), there is a stronger absorption band intensity decrease at the 3600–2800 cm^−1^ region (stretching vibrations of intra- and intermolecular bonds) In conclusion, it can be said that La sorption provides greater amounts of destroyed hydrogen bonds (between hydroxylic groups) in the polymeric chain. 

**Figure 11:**
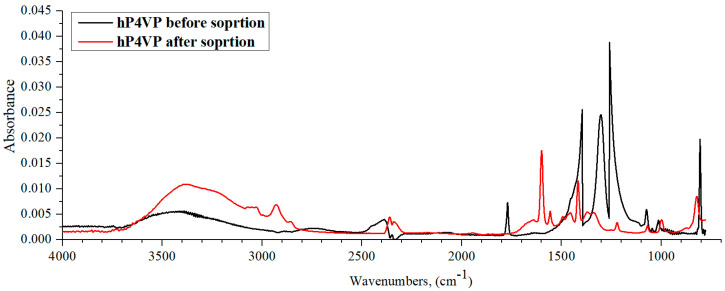
IR spectra of hP4VP before and after lanthanum sorption by interpolymer system 17%hPMAA-83%hP4VP.

IR spectra of hP4VP before and after lanthanum sorption by the studied interpolymer system are shown in Figure 11. Figure 11 shows that the IR spectrum of P4VP after sorption is characterized by the same trend as in the case of the hPAA-hP4VP interpolymer system (Figure 9). Thus, one can observe some absorption bands’ intensities decrease to the level of noises; additionally, new characteristic frequency formations occur. The spectrum of the basic hydrogel after La sorption shows C=N group’s corresponding absorption band, which points to increase in the electron-donating effect. The resulting IR spectrum shows a shift in R_2_C=NH^+^ group absorption band from the region of 2500–2350 cm^−1^ to the region of 2400–2300 cm^−1^. It should also be noted that after the sorption of lanthanum ions, the absorption band intensity of intra- and intermolecular bond vibrations increased, and this change in intensity is much more noticeable in the hPMAA-hP4VP system than for the same polymeric hydrogel (hP4VP) in the interpolymer system hPAA-hP4VP. These facts can confirm the proposed lanthanum ions sorption mechanism by P4VP hydrogel described above. The initial hP4VP presents in lanthanum nitrate in charged state; La sorption provides deionization of the polybasis, wherein there is occurrence of enhancing the electron-donating effect of the pyridine’s ring heteroatom. Consequently intra- and intermolecular bonds formed along with coordination complex of the heteroatom with lanthanum.

#### 3.3.3. Characterization of the IR Spectra of Hydrogels PAA/P2M5VP by Interpolymer System 67%hPAA-33%hP2M5VP

**Figure 12:**
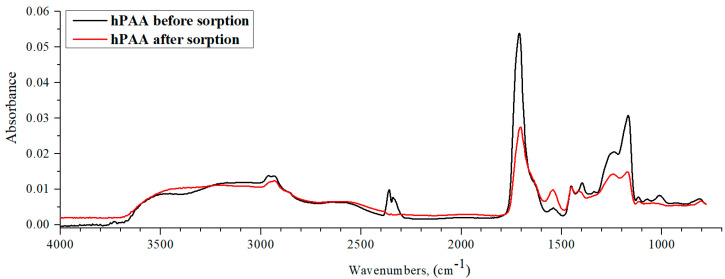
IR spectra of hPAA before and after lanthanum sorption by interpolymer system 67%hPAA-33%hP2M5VP.

Figure 12 shows the IR spectra of hPMAA before and after lanthanum sorption by the studied interpolymer system. From the obtained hPAA IR spectra, it can be seen that the same mechanism of the sorption of lanthanum ions is observed as described above for the hPAA-hP4VP system: through the electrostatic interaction of the negatively charged carboxyl group in the polymer chain and positive lanthanum ions. This fact confirms the COOH group’s absorption band intensity decrease in the region of 1700 cm^−1^ by almost 2 times. It should be noted that this spectrum of PAA hydrogels after sorption is almost identical to the spectrum obtained for the same hydrogel but in the interpolymer system hPAA-hP4VP. The absorption regions of the CH-CO group remained unchanged at 1440–1380 cm^−1^, hydrogen bonds (between -OH fragments) decrease in the composition of the carboxyl group—3600–2900 cm^−1^, and the C-O absorption band intensity decrease in the carboxyl group—1300–1120 cm^−1^.

**Figure 13:**
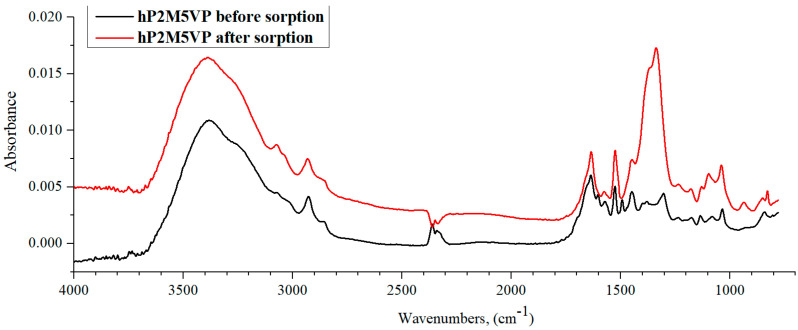
IR spectra of hP2M5VP before and after lanthanum sorption by interpolymer system 67%hPAA-33%hP2M5VP.

IR spectra of hP2M5VP before and after lanthanum sorption by the studied interpolymer system are shown in Figure 13. The polybasis’ absorption band intensity increase indicates the sorption process occurring in the hydrogel. Thus, intensity of the bands increases in the following areas: 3400–3100 cm^−1^ (corresponding to intra-/intermolecular bonds, mainly, associated NH groups); 1700–1480 cm^−1^ (determines the oscillations of the ring); 1560–1480 cm^−1^ (C=N group); and 1150–1000 cm^−1^, which is characteristic only for pyridines. It can also be observed from this figure that after sorption in the spectrum of P2M5VP, the absorption band intensity decreased in 2390–2290 cm^−1^, which corresponds to the R_2_C=NH^+^ group; in addition, the intensity of the characteristic peak at 1310 cm^−1^ is responsible for the C=N bond. It follows from this that deionization of the polybase occurs in the process of sorption. When comparing the IR spectra of hydrogels P2M5VP with P4VP, it can be noted that the spectrum of P2M5VP before sorption differs more strongly from the spectrum of the same polymer after sorption than that of P4VP (Figure 9 and Figure 13). This is due to the location of the heteroatom and double bond in the structure of the pyridine ring with respect to the polymeric backbone. In the structure of P2M5VP, the interaction of a polymer chain with a double bond at a heteroatom in the pyridine ring, due to their close location, has a stronger effect on the stretching and bending vibrations of groups in the polymer. An additional effect is also exerted by the presence of a methyl substituent in the ring in the 2nd position, the characteristic band which is recorded in the 2930–2920 cm^−1^ region in the P2M5VP IR spectrum.

#### 3.3.4. Characterization of the IR Spectra of Hydrogels PMAA/P2M5VP by Interpolymer System 50%hPMAA-50%hP2M5VP

**Figure 14:**
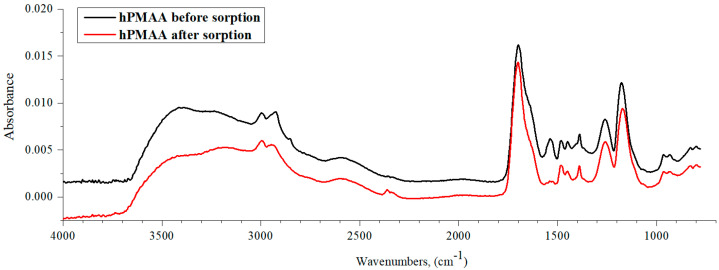
IR spectra of hPMAA before abd after lanthanum sorption by interpolymer system 50%hPMAA-50%hP2M5VP.

Figure 14 shows the IR spectra of hPMAA before and after lanthanum sorption by the studied interpolymer system. The PMAA spectrum contour remained unchanged after La sorption compared to the spectrum of the hydrogel without sorption. Nonetheless, the characteristic peak intensities decreased by over 2 times, which indicates the occurrence of La sorption. The mechanism of La sorption by hPMAA can be characterized by the electrostatic attraction of the metal ions to carboxylic groups (evidenced by –COOH group absorption band intensity decrease in the 1700 cm^−1^ region). Additionally, the difference between the spectrum of PMAA and PAA is wide absorption band intensity, with a clear decrease in the 3600–2800 cm^−1^ region (intra- and intermolecular bonds’ stretching vibrations) owing to the impact of the CH_3_ substituent in the structure of polymethacrylic acid. A comparison of PMAA spectra in interpolymer systems hPMAA-hP4VP and hPMAA-hP2M5VP shows that characteristic absorption band intensities decrease when the presence of hP4VP is higher (points to stronger sorption properties of hPMAA-hP4VP system).

**Figure 15:**
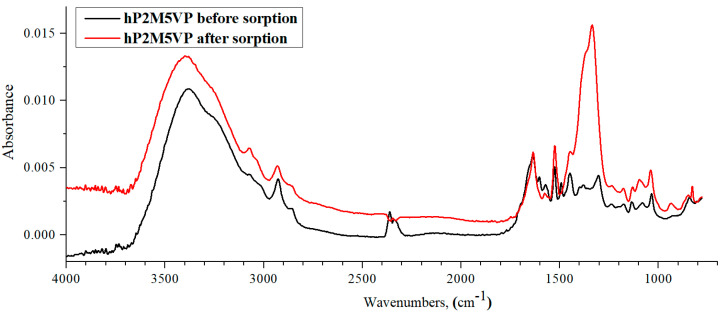
IR spectra of hP2M5VP before and after lanthanum sorption by interpolymer system 50%hPMAA-50%hP2M5VP.

IR spectra of hP2M5VP before and after lanthanum sorption by the studied interpolymer system are shown in Figure 15. The hP2M5VP spectrum absorption band intensity increase points to the La ions sorption. These changes are noticeable in the 3400–3100 cm^−1^ region (intra- and intermolecular bonds—mainly associated NH groups) and the 1170–1050 cm^−1^ region, which is only characteristic to pyridines. As mentioned above, the La sorption process is accompanied by a loss of charges on the polymeric chain; this is evidenced by an absorption band intensity decrease in the 2390–2290 cm^−1^ region (R_2_C=NH^+^ group) and by a characteristic peak’s intensity increase in the 1310 cm^−1^ region (C=N connection).

In the process of sorption, there is a loss of charge on the polymer chain, as evidenced by a decrease in the intensity of the absorption band in the region of 2390–2290 cm^−1^, which corresponds to the R_2_C=NH^+^ group and a sharp increase in the intensity of the characteristic peak in the region of 1310 cm^−1^, which is responsible for the C=N connection. However, it should be noted that in the case of the hPAA-hP2M5VP interpolymer system, the amplitude of changes in such intensities is much higher, which is reflected in the higher sorption capacity of the above system (Figure 13). During La sorption, the following absorption bands are almost remained unchanged: 1700–1480 cm^−1^ (pyridine ring’s vibrations) and 1560–1480 cm^−1^ (C=N bond). Spectrum of hP2M5VP at the 2930–2920 cm^−1^ region shows the impact of the -CH_3_ substituent of the polymeric hydrogel on the structure and sorption properties of the macromolecule. As shown by the IR-spectrometric analysis, the spectra of P2M5VP and P4VP hydrogels are very different, which was described above (Figure 9, Figure 11 and Figure 13) owing to the impact of heteroatom and double-bond locations in pyridine ring relative to the polymeric backbone.

## 4. Conclusions

The obtained results indicate that in the developed interpolymer systems (hPAA-hP4VP, hPMAA-hP4VP, hPAA-hP2M5VP, and hPMAA-hP2M5VP), there is a transition of initial polymeric hydrogels to a highly ionized state. The main difference in the behavior of the polymers is the bulky methyl substituent in the structure of polymeric hydrogels, which leads to slower ionization of both polymers in the interpolymer systems. The remote interaction effect in the interpolymer systems provides significant increase (up to 30%) of the sorption properties of the initial polymeric hydrogels. The obtained results on lanthanum sorption show competitive sorption efficiency in comparison with other methods of lanthanum sorption, such as the application of ion-exchangers [40,41], natural tuffs [42,43], magnetic alginate beads [44], and kaolin composite [45]. Such results are promising to be used as alternative to existing sorbents based on industrial ion-exchangers for effective sorption of REMs. These results are of great importance for the Republic of Kazakhstan due to the fact that there are many ore deposits containing REMs along with the hydrometalurgical leaching solutions containing REM.

## Figures and Tables

**Table 1:** Lanthanum sorption degree of the interpolymer systems.

t, h	η(La), %						
	100%	83%:17%	67%:33%	50%:50%	33%:67%	17%:83%	100%
hPAA:hP4VP, mol.%:mol.%							
0	0	0	0	0	0	0	0
0.5	12.90	29.34	30.77	38.02	43.07	40.38	10.67
1	16.07	30.36	36.99	53.57	56.58	54.91	15.28
2	22.93	43.27	54.77	68.98	73.06	70.65	20.41
6	29.00	60.99	68.15	79.94	84.81	81.43	28.82
24	44.33	71.68	76.22	85.46	89.69	87.22	45.00
48	60.94	84.90	86.71	92.42	94.51	92.98	49.54
hPMAA:hP4VP, mol.%:mol.%							
0	0	0	0	0	0	0	0
0.5	11.74	20.05	22.28	31.61	35.65	38.80	10.67
1	18.92	29.80	31.89	40.11	41.83	45.63	15.28
2	25.68	46.84	49.53	58.21	60.99	63.55	20.41
6	34.08	61.60	64.80	68.65	71.11	74.32	28.82
24	51.67	74.92	77.66	80.82	82.77	85.27	45.00
48	56.34	78.31	83.04	86.53	88.29	90.80	49.54
hPAA:hP2M5VP, mol.%:mol.%							
0	0	0	0	0	0	0	0
0.5	12.90	33.75	38.25	36.48	26.09	21.82	9.08
1	16.07	45.31	53.15	51.81	38.07	30.96	12.99
2	22.93	58.67	68.93	67.26	55.48	47.26	20.41
6	29.00	71.20	80.03	78.27	68.89	61.22	27.71
24	44.33	81.89	88.75	87.22	78.82	75.48	44.76
48	60.94	87.13	91.55	90.24	84.81	81.23	47.74
hPMAA:hP2M5VP, mol.%:mol.%							
0	0.00	0.00	0.00	0.00	0.00	0.00	0.00
0.5	11.74	16.89	30.27	32.77	25.81	18.38	9.08
1	18.92	25.35	41.55	44.19	36.25	30.13	12.99
2	25.68	40.62	56.35	59.04	52.92	46.10	20.41
6	34.08	58.90	69.68	72.60	67.17	62.71	27.71
24	51.67	73.44	81.98	83.98	78.82	74.64	44.76
48	56.34	75.95	87.36	90.10	85.36	81.23	47.74

**Table 2:** Dynamic exchange capacity (in relation to lanthanum) of the interpolymer systems.

t, h	Q(La) mmol/L, %						
	100%	83%:17%	67%:33%	50%:50%	33%:67%	17%:83%	100%
hPAA:hP4VP, mol.%:mol.%							
0	0	0	0	0	0	0	0
0.5	1.17	2.20	2.31	2.85	3.23	3.03	0.80
1	1.46	2.27	2.77	4.02	4.25	4.12	1.15
2	2.09	3.25	4.11	5.18	5.48	5.30	1.53
6	2.64	4.58	5.11	5.99	6.36	6.10	2.17
24	4.04	5.38	5.72	6.41	6.72	6.54	3.38
48	4.32	6.37	6.50	6.93	7.10	6.97	3.71
hPMAA:hP4VP, mol.%:mol.%							
0	0	0	0	0	0	0	0
0.5	0.88	1.51	1.67	2.37	2.68	2.91	0.80
1	1.42	2.23	2.39	3.00	3.14	3.43	1.15
2	1.92	3.52	3.71	4.36	4.57	4.76	1.53
6	2.56	4.62	4.86	5.16	5.33	5.57	2.17
24	3.88	5.62	5.83	6.06	6.20	6.39	3.38
48	4.22	5.88	6.23	6.48	6.62	6.80	3.71
hPAA:hP2M5VP, mol.%:mol.%							
0	0	0	0	0	0	0	0
0.5	1.17	2.53	2.86	2.74	1.95	1.64	0.68
1	1.46	3.40	3.99	3.89	2.85	2.32	0.97
2	2.09	4.40	5.18	5.05	4.16	3.55	1.53
6	2.64	5.35	6.01	5.87	5.17	4.59	2.08
24	4.04	6.14	6.65	6.54	5.91	5.67	3.35
48	4.32	6.53	6.86	6.76	6.36	6.10	3.58
hPMAA:hP2M5VP, mol.%:mol.%							
0	0.00	0.00	0.00	0.00	0.00	0.00	0.00
0.5	0.88	1.27	2.27	2.46	1.93	1.39	0.68
1	1.42	1.90	3.12	3.32	2.72	2.26	0.97
2	1.92	3.05	4.22	4.42	3.97	3.46	1.53
6	2.56	4.42	5.23	5.45	5.05	4.70	2.08
24	3.88	5.51	6.15	6.30	5.92	5.60	3.35
48	4.22	5.70	6.55	6.75	6.40	6.09	3.58

## Data Availability

The data presented in this study are available upon request from the corresponding author.
